# Combined healthy lifestyles and overactive bladder: a cross-sectional study of NHANES 2007–2020

**DOI:** 10.3389/fnut.2025.1603078

**Published:** 2025-07-01

**Authors:** Tianjie Li, Jian Hou, Bo Xiao, Jianxing Li, Xiaodong Liu

**Affiliations:** ^1^Department of Urology, The First Affiliated Hospital of Kunming Medical University, Kunming, China; ^2^Department of Urology, Beijing Tsinghua Changgung Hospital, Beijing, China

**Keywords:** overactive bladder, lifestyle score, NHANES, physical activity, smoking, alcohol consumption, diet, waist circumference

## Abstract

**Objectives:**

The relationship between adherence to a combined healthy lifestyle and the risk of overactive bladder (OAB) remains unclear. This study aimed to investigate the association between a composite healthy lifestyle score and risk of OAB in a nationally representative sample of adults.

**Methods:**

This cross-sectional study utilized data from 20,195 non-pregnant adults aged 20–79 years in the National Health and Nutrition Examination Survey (NHANES) 2007–2020. A healthy lifestyle score was constructed based on five components: current non-smoking, low-to-moderate alcohol consumption, adequate physical activity, a healthy diet, and optimal waist circumference. OAB was defined using self-reported urinary urgency incontinence and nocturia symptoms. Weighted multivariable logistic regression models were employed to assess the association between the healthy lifestyle score and risk of OAB, adjusting for demographic, socioeconomic, and clinical covariates.

**Results:**

Among the 20,195 participants, 3,901 (14.58%) were identified as having OAB. A higher HLS was inversely associated with risk of OAB in a dose–response manner. Compared with individuals having 0–1 healthy lifestyle factors, those with 4–5 factors had a 46% lower risk of OAB (adjusted OR: 0.54, 95% CI: 0.45–0.65). Each additional healthy lifestyle factor was associated with a 17% lower risk of OAB (OR: 0.83, 95% CI: 0.79–0.88). Sensitivity analyses confirmed the robustness of these associations. Among individual components, non-smoking, moderate alcohol intake, regular physical activity, a healthy diet, and optimal waist circumference were each independently associated with a lower risk of OAB.

**Conclusion:**

Adherence to a combination of healthy lifestyle behaviors was significantly associated with a lower risk of OAB. These findings emphasize the potential role of lifestyle-based interventions in OAB prevention and management. Given the rising prevalence of OAB, particularly in aging populations, incorporating lifestyle modifications into clinical and public health strategies may offer an effective, non-pharmacological approach to mitigating risk of OAB. Further longitudinal studies are warranted to establish causality and elucidate the underlying biological mechanisms.

## 1 Introduction

Overactive bladder (OAB) is a prevalent and debilitating urological condition characterized by urgency, often accompanied by increased urinary frequency, nocturia, and urgency urinary incontinence (UUI) (1). OAB affects a substantial proportion of the global population, significantly impairing quality of life and contributing to psychological distress, including anxiety and depression (2, 3). The prevalence of OAB has been rising, particularly among older adults (4, 5). With the progressive aging of the global population, OAB presents increasing challenges to healthcare systems. In the United States, OAB has been shown to incur billions of dollars in healthcare expenditures annually, with per capita healthcare costs of OAB patients exceeding those of comparable individuals without OAB by more than 2.5 times (6, 7). However, its precise etiology remains incompletely understood, with emerging evidence suggesting that detrusor overactivity, autonomic nervous system dysfunction, metabolic syndrome, sex hormone deficiency and urinary microbiota may contribute to its pathogenesis (8).

Lifestyle modifications are widely recognized as a cornerstone in the prevention and management of various chronic diseases, including cardiovascular disease, diabetes, and cancer (9–11). However, the association between combined healthy lifestyle factors and the risk of OAB remains largely unexplored. Previous studies have predominantly examined individual lifestyle factors in relation to OAB. A significant association has been identified between both high body mass index (BMI) (12, 13) and increased waist circumference (14, 15)with a heightened risk of OAB. Similarly, smoking, alcohol consumption, physical activity, and dietary habits have also been investigated in relation to risk of OAB (16–21). However, behavioral factors are often interrelated, and individuals tend to adopt lifestyle patterns that encompass multiple factors (22). Therefore, lifestyle factors should be analyzed collectively to better assess their overall health impact (23). Despite this, existing studies have not systematically evaluated the cumulative effect of multiple healthy lifestyle behaviors, leaving a critical gap in understanding their combined influence on risk of OAB.

In light of this, the present study explores the relationship between a composite healthy lifestyle score, which incorporates smoking status, alcohol consumption, physical activity, dietary habits, and body weight, and the risk of OAB using data from the National Health and Nutrition Examination Survey (NHANES). By analyzing a nationally representative dataset, we aim to provide insights into the potential protective role of adhering to a combination of healthy lifestyle behaviors in mitigating risk of OAB, contributing to a better understanding of preventive strategies for this prevalent condition.

## 2 Methods

### 2.1 Study population

**Figure 1:**
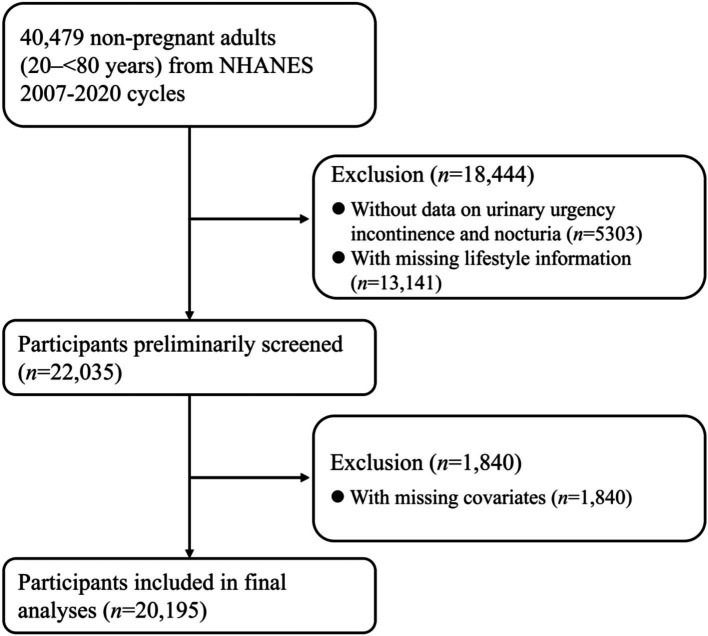
Flow of eligible participants selection. NHANES, National Health and Nutrition Examination Survey.

Nation Health and Nutrition Examination Survey (NHANES), a nationally representative program of surveys designed to assess the health and nutritional status of adults and children in US, examined approximately 5,000 non-institutional civilians each year. NHANES was approved by the National Center for Health Statistics (NCHS) Ethics Review Board and had obtained informed consents from participants. We selected 40,479 non-pregnant adults (aged 20- < 80 years) from NHANES 2007–2020 cycles for eligibility screening. A total of 22,035 adults were left after excluding 5,303 subjects without data on urinary urgency incontinence and nocturia and 13,141 subjects with missing lifestyle information. Moreover, 1,840 subjects with missing covariates were excluded. Missing numbers and percentages of covariates were shown in Supplementary Table S1. Finally, the current cross-sectional study comprised 20,195 non-pregnant adults, and the flow of eligibility screening was shown in Figure 1.

### 2.2 Construction of healthy lifestyle score

Healthy lifestyle score was constructed by summing the number of healthy lifestyle factors, including current nonsmoking, low-to-moderate alcohol drinking, adequate physical activity, healthy diet, and optimal waist circumference, according to the method by Zhang et al. (24). The healthy lifestyle score, whose higher values indicated healthier lifestyles, ranged from 0 to 5. Definitions of healthy levels of lifestyle factors were shown in Supplementary Table S2.

### 2.3 Definition of overactive bladder

As defined by the International Continence Society, urinary urgency incontinence and nocturia are the main features of overactive bladder (OAB), and their evaluation constitutes the assessment of OAB. Participants were asked three questions: “During the past 12 months, {have you/has SP} leaked or lost control of even a small amount of urine with an urge or pressure to urinate and {you/he/she} could not get to the toilet fast enough?,” “How frequently does this occur?,” and “During the past 30 days, how many times per night did {you/SP} most typically get up to urinate, from the time {you/s/he} went to bed at night until the time {you/he/she} got up in the morning?.” The scoring details were explained in Supplementary Table S3, and a score of three or above indicates OAB.

### 2.4 Assessment of covariates

Demographic, socioeconomic, lifestyle information was collected with a computer-assisted personal interview system by trained interviewers. Race/ethnicity was categorized as non-Hispanic White, non-Hispanic Black, Mexican American, and others. Marital status was categorized as married, single (widowed, divorced, separated, never married), and living with partner. Self-reported education attainment was grouped as under high school, high school, and above high school. Family poverty-income ratio (PIR), which was calculated by dividing family (or individual) income by the poverty guidelines specific to the survey year, was applied to measure income status. Blood pressure measurements were collected by examiners with mercury sphygmomanometers. Hypertension was defined as systolic blood pressure ≥ 140 mmHg, diastolic blood pressure ≥90 mmHg, physician-diagnosed hypertension, or currently taking prescribed medicine for hypertension. Diabetes was defined as fasting plasma glucose (FPG) ≥ 126 mg/dL, glycated hemoglobin A1c (HbA1c) ≥ 6.5%, oral glucose tolerance test (OGTT) two-hour glucose ≥ 200 mg/dL, self-reported physician-diagnosed diabetes, use of insulin or oral hypoglycemic medication.

### 2.5 Statistical methods

Taking unequal probability of selection and over sampling of certain subpopulations into account, we applied sampling weights, strata, and primary sampling units in analyses. Continuous variables were expressed with weighted means and standard errors (SEs), and categorical variables were expressed with numbers and weighted percentages. Means and proportions of population characteristics were compared with linear regression for continuous variables and logistic regression for categorical variables.

Weighted multivariable logistic regression model was used to examine the association of healthy lifestyle score with risk of OAB. In multivariable model 1, we adjusted for age (<50, ≥50 years), sex (male, female), and race/ethnicity (non-Hispanic White, others). In multivariable model 2, we further adjusted for marital status (married, others), family PIR (<3.5, ≥3.5), education attainment (above high school, high school and below), hypertension (yes, no), and diabetes (yes, no). We also calculated multivariable-adjusted OR with 95% CI for OAB associated with each additional healthy lifestyle factor. To examine whether the aforementioned confounders modified the association of healthy lifestyle score with risk of OAB, we performed stratified and multiplicative interaction analyses. To examine the contributions of different lifestyle factors, we first assessed the associations of five lifestyle factors with OAB, with all lifestyle factors mutually adjusted for. Then, we reconstructed new healthy lifestyle scores by removing one lifestyle factor each time from the score and adjusted the removed factor in the models.

The following sensitivity analyses were conducted to evaluate the robustness of our results. First, we redefined the healthy level of alcohol drinking as none or low-to-moderate alcohol drinking (≤28/14 g/day for men/women). Second, propensity score (PS) adjustment was applied to cope with observed confounding. Third, missing covariates were imputed under the missing at random assumption using multiple imputation (MI) with fully conditional specification (FCS) and random forest method. Fourth, we calculated an assessment of potential residual confounding with E-values, defined as the minimum strength of association on the OR scale that an unmeasured confounder would need to have with both the exposure and the outcome to fully explain away the observed exposure-outcome association, conditional on the measured covariates (25, 26). Finally, we constructed a weighted healthy lifestyle score to better reflect the effect of each healthy lifestyle factor on the outcome. A score of 1 was assigned to a healthy lifestyle, otherwise 0 was assigned. Weighted standardized healthy lifestyle score was calculated based on *β* coefficients of each lifestyle in the logistic regression model with all 5 lifestyle factors and adjustment for all covariates. Each binary lifestyle factor was multiplied by the β coefficients, summed, divided by the sum of the β coefficients, and multiplied by 5. The weighted score, ranging from 0 to 5, considers the magnitudes of the adjusted odds ratio (OR) for each lifestyle in each lifestyle pattern as a combination of 5 lifestyle factors. We then categorized the weighted lifestyle scores into quartiles to avoid extreme groups. Restricted cubic spline (RCS) with 3 knots was further plotted to visualize the dose–response relationship between weighted healthy lifestyle score and OAB.

## 3 Results

### 3.1 Population characteristics

This cross-sectional study comprised 20,195 non-pregnant US adults (weighted mean age 46.77 years and 50.66% male), of which 3,901 (14.58%) OAB cases were ascertained (Table 1). The prevalence of current nonsmoking, low-to-moderate alcohol drinking, adequate physical activity, healthy diet, and optimal waist circumference were 79.33, 74.57, 40.49, 41.02, and 22.94%, respectively (Table 1). Population characteristics across healthy lifestyle scores were displayed in Supplementary Table S4. Participants with more healthy lifestyle factors were more likely to be male and younger, non-Hispanic White, married, well-educated, had better income status, and had higher HEI-2015 score and lower values of BMI and waist circumference (all *p* < 0.001) (Supplementary Table S4). Moreover, those with fewer healthy lifestyle factors were more likely to be hypertensive and diabetic (all *p* < 0.001) (Supplementary Table S4).

**Table 1:** Characteristics of study participants.

Characteristics^a^	Overall (*N* = 20,195)
Age, years	46.77 (0.25)
BMI, kg/m^2^	29.22 (0.09)
Waist circumference, cm	99.95 (0.23)
HEI-2015	51.22 (0.20)
Male, *n* (%)	10,451 (50.66)
Race/ethnicity, *n* (%)	
Non-Hispanic White	8,898 (70.97)
Non-Hispanic Black	4,535 (10.13)
Mexican American	2,735 (7.39)
Others	4,027 (11.52)
Marital status, *n* (%)	
Married	10,948 (58.57)
Single	7,924 (34.70)
Living with a partner	1,323 (6.73)
Education attainment, *n* (%)	
Under high school	3,911 (12.60)
High school	4,587 (22.14)
Above high school	11,697 (65.26)
Family PIR, *n* (%)	
<1.3	5,925 (18.84)
1.3- < 3.5	7,417 (33.93)
≥3.5	6,853 (47.22)
Current nonsmoking, *n* (%)	15,638 (79.33)
Low-to-moderate alcohol drinking, *n* (%)	14,527 (74.57)
Adequate physical activity, *n* (%)	7,232 (40.49)
Healthy diet, *n* (%)	8,078 (41.02)
Optimal waist circumference, *n* (%)	4,657 (22.94)
No. of healthy lifestyle factors, *n* (%)	
0	705 (3.22)
1	3,442 (15.03)
2	6,286 (29.60)
3	5,774 (29.63)
4	3,144 (17.43)
5	844 (5.11)
Hypertension, *n* (%)	8,371 (36.51)
Diabetes, *n* (%)	3,433 (12.48)
OAB, *n* (%)	3,901 (14.58)

^a^ Continuous variables were expressed as weighted means and standard errors and categorical variables were expressed as numbers and weighted percentages. The sums of percentages may not reach 100%, owing to the rounding of decimals and missing values. BMI, body mass index; HEI, Healthy Eating Index; OAB, overactive bladder; PIR, poverty-income ratio.

### 3.2 Association of healthy lifestyle score with OAB

In crude model, the OR for OAB comparing participants with 4–5 vs. 0–1 healthy lifestyle factors was 0.30 (95% CI: 0.26–0.36) (Table 2). After adjusting for all covariates, adults with 4–5 healthy lifestyle factors were confronted with 46% (OR: 0.54, 91% CI: 0.45–0.65) lower risk of OAB as compared with those with 0–1 healthy lifestyle factors (Table 2). In addition, each additional healthy lifestyle factor was associated with 17% (OR: 0.83, 95% CI: 0.79–0.88) decreased risk of RA (Table 2).

**Table 2:** Association of healthy lifestyle score with risk of OAB.

Variable	No. of healthy lifestyle factors				Each additional healthy lifestyle factor
	0–1	2	3	4–5	
Case/total (%)	1169/4147 (28.19)	1370/6286 (21.79)	948/5774 (16.42)	414/3988 (10.38)	
Crude model	1.00 (reference)	0.65 (0.56–0.77)	0.47 (0.40–0.56)	0.30 (0.26–0.36)	0.71 (0.68–0.74)
Model 1^a^	1.00 (reference)	0.63 (0.54–0.74)	0.47 (0.39–0.56)	0.34 (0.29–0.41)	0.73 (0.69–0.77)
Model 2^b^	1.00 (reference)	0.74 (0.62–0.87)	0.62 (0.52–0.73)	0.54 (0.45–0.65)	0.83 (0.79–0.88)

^a^ Model 1 was adjusted for age (<50, ≥50 years), sex (male, female), and race/ethnicity (non-Hispanic White, others). ^b^ Model 2 was further adjusted for marital status (married, others), family poverty-income ratio (<3.5, ≥3.5), education attainment (above high school, high school and below), hypertension (yes, no), and diabetes (yes, no). OAB, overactive bladder.

### 3.3 Stratified, interaction, and sensitivity analyses

To examine whether the association between healthy lifestyle score and risk of OAB differed by age, sex, race/ethnicity, marital status, family PIR, education attainment, hypertension, and diabetes, we performed stratified and interaction analyses (Table 3). The inverse association between healthy lifestyle score and OAB persisted in all subgroups and seemed to be stronger in those aged <50 years and with family PIR < 3.5 (both P-interaction<0.05).

**Table 3:** Association of healthy lifestyle score with OAB stratified by confounders.

Confounders	No. of subjects	OR (95% CI)a	P-interaction
Overall	20,195	0.83 (0.79–0.88)	
Age, years			<0.001
<50	10,521	0.77 (0.72–0.83)	
≥50	9,674	0.88 (0.82–0.94)	
Sex			0.06
Male	10,451	0.89 (0.82–0.96)	
Female	9,744	0.80 (0.75–0.86)	
Race/ethnicity			0.21
Others	11,297	0.81 (0.76–0.86)	
Non-Hispanic White	8,898	0.85 (0.79–0.91)	
Family PIR			0.04
<3.5	13,342	0.79 (0.74–0.85)	
≥3.5	6,853	0.90 (0.82–0.99)	
Education attainment			0.50
High school and below	8,498	0.81 (0.74–0.89)	
Above high school	11,697	0.86 (0.80–0.92)	
Hypertension			0.14
No	11,824	0.81 (0.75–0.87)	
Yes	8,371	0.87 (0.81–0.94)	
Diabetes			0.94
No	16,762	0.84 (0.79–0.89)	
Yes	3,433	0.82 (0.75–0.90)	

^a^ Data were presented as odds ratio (95% confidence interval) associated with each additional healthy lifestyle factor. Models were adjusted for age (<50, ≥50 years), sex (male, female), and race/ethnicity (non-Hispanic White, others), marital status (married, others), family poverty-income ratio (<3.5, ≥3.5), education attainment (above high school, high school and below), hypertension (yes, no), and diabetes (yes, no). CI, confidence interval; OAB, overactive bladder; OR, odds ratio.

As for individual lifestyle factors, current nonsmoking, low-to-moderate drinking, adequate physical activity, healthy diet, and optimal waist circumference were associated with OAB with ORs (95% CIs) of 0.82 (0.70–0.95), 0.81 (0.71–0.92), 0.89 (0.79–1.00), 0.87 (0.78–0.98), and 0.71 (0.61–0.83), respectively (Supplementary Table S5). The associations of four-component lifestyle scores with OAB were attenuated when current nonsmoking, low-to-moderate drinking, adequate physical activity, healthy diet, and optimal waist circumference were removed from the score, with ORs (95% CIs) comparing 3–4 vs. 0–1 healthy lifestyle factors being 0.67 (0.58–0.78), 0.69 (0.59–0.81), 0.62 (0.54–0.70), 0.62 (0.52–0.72), and 0.62 (0.53–0.72), respectively (Supplementary Table S6).

**Figure 2:**
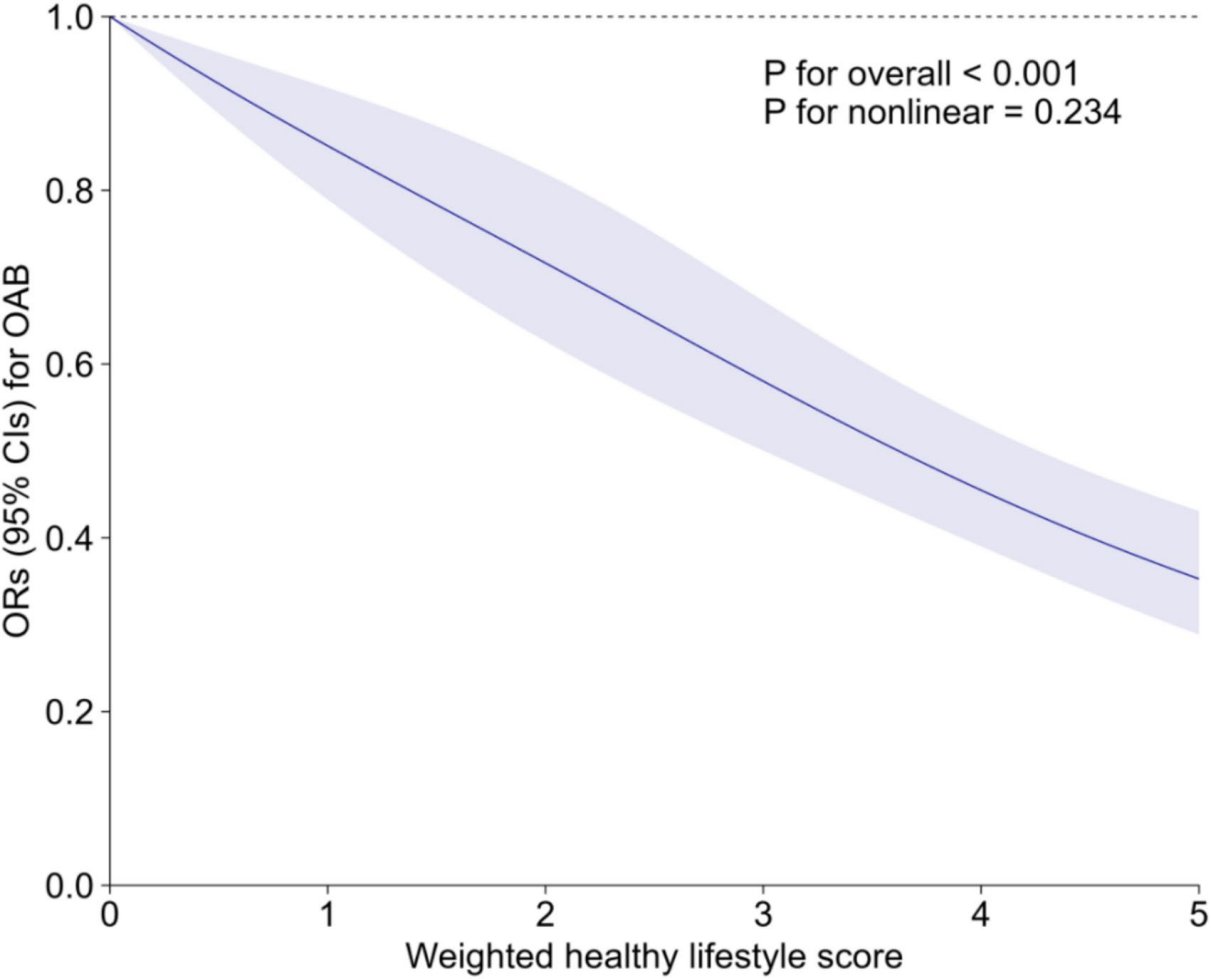
Association of weighted healthy lifestyle score with risk of OAB. Line represents multivariable-adjusted OR, and shaded area represents 95% CI. Models were adjusted for age (<50, ≥50 years), sex (male, female), race/ethnicity (non-Hispanic White, others), marital status (married, others), family poverty-income ratio (<3.5, ≥3.5), education attainment (above high school, high school and below), hypertension (yes, no), and diabetes (yes, no). CI, confidence interval; OAB, overactive bladder; OR, odds ratio.

Several sensitivity analyses were performed to evaluate the robustness of our results (Supplementary Tables S7–S12). The inverse association persisted in sensitivity analysis redefining the healthy level of alcohol drinking (Supplementary Table S7), with OR (95% CI) for OAB associated with each additional healthy lifestyle factor being 0.85 (0.81–0.90). Applying PS adjustment to cope with observed confounders, the OR for OAB comparing participants with 4–5 vs. 0–1 healthy lifestyle factors was 0.53 (95% CI: 0.45–0.63) (Supplementary Table S8). Additionally, the observed significant inverse association of healthy lifestyle score with OAB was not negated by inputting missing covariates with MI (Supplementary Table S9). The E-value was 3.11 (Supplementary Table S10), suggesting that it would take very strong confounding to negate the inverse association observed in our study. Finally, a weighted healthy lifestyle score was constructed to better reflect the effect of each healthy lifestyle factor on the outcome. As shown in Supplementary Table S11, optimal waist circumference contributed most to the weighted healthy lifestyle score (weighted *β* = 0.34), followed by low-to-moderate drinking (weighted *β* = 0.21),current nonsmoking (weighted *β* = 0.20), healthy diet (weighted *β* = 0.14), and adequate physical activity (weighted *β* = 0.12). After adjusting for all covariates, adults with the highest weighted healthy lifestyle score quartile were confronted with 50% (OR: 0.50, 95% CI: 0.41–0.60) lower risk of OAB as compared with those with the lowest weighted healthy lifestyle score quartile (Supplementary Table S12). Inverse linear dose–response relationship between weighted healthy lifestyle score and OAB was depicted in the RCS (P-overall<0.001, P-nonlinearity = 0.234) (Figure 2).

## 4 Discussion

Previous research has primarily focused on the individual effects of lifestyle factors such as smoking, physical activity, diet, and obesity on overactive bladder (OAB) risk. Our study adds to this literature by examining the combined influence of multiple healthy lifestyle behaviors, demonstrating a dose-dependent inverse relationship between adherence to a composite healthy lifestyle score and OAB risk. These findings underscore the potential benefits of a holistic lifestyle approach, which aligns with evidence from other chronic diseases where combined lifestyle modifications have shown superior preventive effects compared to individual factors alone (27–30).

In this large-scale cross-sectional study using data from NHANES, we identified a strong inverse association between adherence to a combined healthy lifestyle (CHL) score and the risk of OAB. Individuals adhering to ≥4 healthy lifestyle factors exhibited a 46% lower risk of OAB compared to those with ≤1 factor, and each additional healthy lifestyle factor conferred a 17% risk reduction. These findings support the role of modifiable behavioral factors in OAB prevention and management.

Tobacco smoking has long been recognized as a significant risk factor for bladder dysfunction and OAB. In this study, current smoking was associated with a higher risk of OAB. The mechanisms through which smoking contributes to OAB are multifactorial, including neurogenic stimulation (mediated by nicotine’s modulation of the sympathetic nervous system) and direct urothelial irritation (31). Additionally, smoking-induced atherosclerosis and vascular injury may impair bladder perfusion, exacerbating detrusor dysfunction, while smoking-related reductions in testosterone levels have also been suggested as potential contributing factors. (32–34). Given these effects, smoking cessation should be prioritized as an essential component of OAB prevention and management.

The relationship between alcohol consumption and OAB remains complex, with some studies reporting a positive correlation between excessive alcohol consumption and increased urgency or frequency of urination (19, 35), while others report no significant association (20, 36, 37). In our study, individuals who consume alcohol within healthy limits were found to have a lower risk of OAB compared to those who exceed these limits. This suggests that moderate alcohol intake may not significantly contribute to the development of OAB and may even have some cardiovascular protective effects. Although the precise effects of alcohol on bladder tissue remain unclear, urothelial cells are particularly vulnerable to damage due to their direct exposure to alcohol and its metabolites (38). Furthermore, excessive alcohol consumption may increase the risk of bladder irritation, urgency, and frequency, possibly due to its pro-inflammatory effects and the influence of alcohol on the nervous system (39).

Only a few studies have examined the relationship between regular physical activity and OAB, with some suggesting that physical activity may have a beneficial effect on OAB (21, 40). However, the precise mechanisms underlying this relationship remain unclear. In contrast, pelvic floor muscle training has been consistently shown to benefit OAB and is recommended as part of treatment strategies (41–43). Our study found that adherence to the recommended levels of physical activity was associated with a significantly lower risk of OAB. We propose that regular physical activity may improve bladder control by increasing bladder blood flow, enhancing metabolic function, and reducing systemic inflammation, which is known to play a role in many chronic conditions, including OAB (44, 45). Additionally, physical activity appears to help reduce obesity, a known risk factor for OAB. These findings reinforce the importance of exercise as part of a comprehensive OAB management plan, especially considering its benefits in reducing obesity and enhancing pelvic floor strength.

Diet plays an important role in the pathogenesis and symptom modulation of overactive bladder (OAB). A healthy dietary pattern, such as that reflected by a higher Healthy Eating Index-2015 (HEI-2015) score, has been associated with reduced systemic inflammation, improved metabolic health, and better bladder function (46). In our study, participants in the top two quintiles of HEI-2015 score showed significantly lower odds of having OAB, underscoring the potential protective association of high-quality diets. While our analysis did not isolate the effects of individual components, these adequacy components are generally considered beneficial for bladder and metabolic health. In contrast, dietary patterns characterized by low fiber intake and high consumption of processed foods, which are typically reflected by lower HEI-2015 scores, may contribute to adverse physiological processes such as elevated intra-abdominal pressure, chronic systemic inflammation, and alterations in gut microbiota composition, all of which have been potentially implicated in the pathophysiology and symptom exacerbation of OAB (18). Although current evidence does not identify specific HEI-2015 components as causally linked to OAB, our findings support the inclusion of dietary quality assessment in OAB-related public health strategies. Future prospective studies are warranted to clarify the contribution of individual dietary components and validate their potential role in targeted nutritional interventions.

Waist circumference (WC), as an indicator of visceral fat accumulation (47, 48), was strongly associated with risk of OAB in our study. Increased WC correlates with greater abdominal and bladder pressure, which can lead to chronic bladder irritation, detrusor overactivity, and increased urgency symptoms (14). These results underscore the importance of weight management through physical activity and dietary modifications to reduce risk of OAB. Visceral fat, particularly around the abdominal area, is known to exacerbate OAB symptoms through its inflammatory effects and by increasing intra-abdominal pressure, which puts additional stress on the bladder and detrusor muscles (49, 50). Individuals with abdominal obesity may particularly benefit from interventions aimed at reducing visceral fat and improving metabolic health.

We observed significant interactions between age and poverty-income ratio (PIR) with respect to the association between healthy lifestyle adherence and the risk of OAB. Previous studies have indicated that the prevalence of OAB tends to increase with advancing age, potentially due to age-related changes in bladder function and neurodegenerative processes (4). In contrast, individuals with lower PIR have been linked to an increased prevalence of risk factors such as smoking, unhealthy diet, and greater exposure to occupational or environmental stressors, which may elevate OAB susceptibility (51). These observations highlight the potential importance of developing targeted preventive and management strategies for specific subgroups, such as younger individuals and those from lower-income backgrounds.

In conclusion, adherence to a combined healthy lifestyle—incorporating tobacco smoking cessation, moderate alcohol consumption, regular physical activity, a healthy diet, and optimal waist circumference—is associated with a lower risk of OAB. These findings support the importance of modifiable lifestyle factors in OAB prevention. Promoting healthy lifestyle habits, particularly in aging populations, could significantly reduce OAB’s burden on individuals and healthcare systems. Furthermore, for individuals with refractory OAB, lifestyle interventions may offer better long-term outcomes than traditional pharmacological treatments. Future research should focus on longitudinal studies to confirm causal relationships between healthy lifestyles and OAB, and explore the mechanisms through which these interventions impact bladder function.

This study has several key strengths. First, utilizing data from the National Health and Nutrition Examination Survey (NHANES), a large, nationally representative sample, enhances the generalizability of the findings to the broader U. S. adult population. Second, instead of examining individual lifestyle factors separately, this study employed a combined healthy lifestyle approach, allowing for a more comprehensive assessment of how multiple health behaviors collectively influence risk of OAB. Health behaviors are multifaceted and encompass multiple dimensions; therefore, adopting a combined healthy lifestyle pattern analysis provides a more holistic evaluation of their cumulative impact compared to analyzing single risk factors in isolation. In this study, the association between combined healthy lifestyles and reduced risk of OAB was stronger than that observed for individual lifestyle factors, reinforcing the importance of assessing lifestyle patterns as a whole in risk assessment. Third, we applied rigorous statistical controls for key demographic and clinical variables, minimizing potential confounding and strengthening the internal validity of our results. Consistent findings across different cohorts with diverse characteristics and subgroups further consolidate the generalizability of our findings, ensuring their applicability to a broad population.

It is important to note that the cut-offs used to define healthy lifestyle factors in our study—including thresholds for alcohol consumption and waist circumference—are primarily derived from general health guidelines such as those from the WHO and other authoritative bodies. While these cut-offs have been widely applied and are supported by evidence in the context of overall health and metabolic disease prevention, their specific relevance to bladder health and OAB remains less well-defined. The precise thresholds that optimally relate to bladder outcomes require further investigation. Future studies that specifically examine the appropriateness of these cut-offs for OAB and related urinary conditions would help refine prevention strategies and clinical recommendations.

Despite its strengths, this study has several limitations. First, its cross-sectional design limits the ability to establish causal relationships between combined healthy lifestyle factors and OAB risk. Although statistically significant inverse associations were observed, reverse causality cannot be excluded, as individuals with OAB symptoms may be less likely to engage in healthy lifestyle behaviors. Longitudinal or interventional studies are needed to confirm the directionality of these associations. Second, lifestyle factors like smoking, alcohol consumption, physical activity, and diet were self-reported, which may introduce recall bias and inaccuracies. Third, OAB diagnosis was based on self-reported symptoms rather than objective clinical evaluations, potentially leading to misclassification or overestimation of OAB prevalence. The absence of data on OAB severity and specific subtypes further limits our understanding of the condition. Future research integrating clinical evaluations or linking with medical records could improve diagnostic accuracy and provide a more nuanced understanding of OAB. Additionally, while key confounders were controlled for, there may still be unmeasured residual confounding from factors such as genetics, mental health, medications, comorbidities, and environmental exposures. Future studies with more comprehensive assessments of these variables could clarify their impact. Moreover, the results from complete case analyses may be biased due to missing data not being completely at random (MCAR). Lastly, we did not adjust for multiple testing in subgroup analyses, so these results should be considered exploratory. Finally, the composite healthy lifestyle score used in this study aggregates various factors, which may obscure the differential effects of each component. While NHANES data are representative of the U.S. adult population, these results may not be directly applicable to other populations with different cultural or environmental factors.

This study suggests that lifestyle-based interventions targeting smoking cessation, moderate alcohol consumption, physical activity, balanced diet, and optimal waist circumference could reduce OAB risk. Public health strategies should raise awareness about the importance of these behaviors and promote programs for physical activity, dietary improvements, and weight management. Clinically, healthcare professionals might incorporate lifestyle counseling into care, providing personalized recommendations for individuals at higher OAB risk. However, challenges in implementation may arise from varying access to healthcare, socioeconomic barriers, and patient adherence. Future research should evaluate the feasibility, effectiveness, and cost-effectiveness of these interventions across diverse populations to better understand their potential in reducing the healthcare burden of OAB.

## 5 Conclusion

In conclusion, our study demonstrates that adherence to a combined healthy lifestyle, which includes tobacco smoking cessation, moderate alcohol consumption, regular physical activity, a healthy diet, and the maintenance of an optimal waist circumference, is associated with a lower risk of OAB. These findings emphasize the role of modifiable lifestyle factors in OAB prevention and management.

Future research should focus on confirming the causal relationships between combined healthy lifestyle factors and OAB through longitudinal studies, while also investigating the optimal combination and intensity of lifestyle modifications. Further exploration of the underlying biological mechanisms, including inflammation, oxidative stress, and the gut-bladder axis, is essential to better understand their potential roles in OAB pathophysiology. Additionally, evaluating the feasibility and effectiveness of lifestyle interventions through clinical trials will provide valuable insights into their practical applications and integration into multidisciplinary care pathways, which could help inform more sustainable and precision-oriented approaches to OAB management.

## Data Availability

The original contributions presented in the study are included in the article/Supplementary material, further inquiries can be directed to the corresponding author/s.
