# Supplementary material for: Combined healthy lifestyles and overactive bladder: a cross-sectional study of NHANES 2007–2020
Source: Front Nutr. 2025 Jul 1;12:1603078. doi: 10.3389/fnut.2025.1603078 (PMC12256703; doi:10.3389/fnut.2025.1603078)
Supplement: Supplementary file 1 [file Data_Sheet_1.docx]

**Supplementary Materials**

**Table of Contents**

[Table S1. Missing counts and proportions of covariates. 2](#_Toc2068566159)

[Table S2. Definitions of healthy and unhealthy lifestyle factors. 3](#_Toc778673030)

[Table S3. OAB symptom frequency score conversion method. 4](#_Toc392270392)

[Table S4. Characteristics of participants with different numbers of healthy lifestyle factors from the National Health and Nutrition Examination Survey. 5](#_Toc113682054)

[Table S5. Associations of each healthy lifestyle factor with risk of OAB. 7](#_Toc1541319395)

[Table S6. Associations of different lifestyle scores consisting of four lifestyle scores with risk of OAB. 8](#_Toc2007321651)

[Table S7. Association of healthy lifestyle score with risk of OAB after redefining the healthy level of alcohol drinking. 9](#_Toc86893987)

[Table S8. Association of healthy lifestyle score with risk of OAB after propensity score adjustment. 10](#_Toc138359549)

[Table S9. Association of healthy lifestyle score with risk of OAB after imputing missing covariates with multiple imputation. 11](#_Toc1831633989)

[Table S10. E-values and lower limit of 95% CIs for the association of healthy lifestyle score with risk of OAB. 12](#_Toc94373378)

[Table S11. The coefficients of each healthy lifestyle factor in the logistic regression model. 13](#_Toc1290432560)

[Table S12. Association of weighted healthy lifestyle score with risk of OAB. 14](#_Toc862684867)

# Table S1. Missing counts and proportions of covariates.

| **Covariates** | **No. of missing** | **Missing proportion (%)** |
| --- | --- | --- |
| Age | 0 | 0.00 |
| Sex | 0 | 0.00 |
| Race/ethnicity | 0 | 0.00 |
| Marital status | 11 | 0.05 |
| Education attainment | 12 | 0.05 |
| Family PIR | 1824 | 8.28 |
| Hypertension | 1 | 0.00 |
| Diabetes | 0 | 0.00 |

Abbreviations: PIR, poverty-income ratio.

# Table S2. Definitions of healthy and unhealthy lifestyle factors.

| **Factor** | **Healthy level** | **Unhealthy level** |
| --- | --- | --- |
| Tobacco smoking | Current nonsmoking | Current smoking |
| Alcohol drinking | Men: 1-28 g/day; women: 1-14 g/day | Men: 0 or >28 g/day; women: 0 or >14 g/day |
| Physical activity | Moderate-to-vigorous leisure-time physical activity of ≥150 min/week | Moderate-to-vigorous leisure-time physical activity of <150 min/week |
| Diet | Top two-fifths of HEI-2015 score | Bottom three-fifths of HEI-2015 score |
| Waist circumference | Waist circumference <94 cm and <80 cm for men and women, respectively | Waist circumference ≥94 cm and ≥80 cm for men and women, respectively |

Abbreviation: HEI, Healthy Eating Index.

# Table S3. OAB symptom frequency score conversion method.

| **NHANES self-reported symptoms of OAB** | **OABSS score** |
| --- | --- |
| **Frequency of urinary incontinence** | **Urinary incontinence score** |
| Never | 0 |
| <Once per week | 1 |
| >Once per week | 2 |
| Every day or night | 3 |
| **Nocturia frequency** | **Nocturia score** |
| Never | 0 |
| Once per night | 1 |
| Twice per night | 2 |
| ≥3 times per night | 3 |

Abbreviations: OAB, overactive bladder; OABSS, overactive bladder symptom score.

# Table S4. Characteristics of participants with different numbers of healthy lifestyle factors from the National Health and Nutrition Examination Survey.

| **Characteristics^a^** | **No. of healthy lifestyle factors** | | | | ***P*-value^b^** |
| --- | --- | --- | --- | --- | --- |
|  | **0-1** | **2** | **3** | **4-5** |  |
| No. of participants | 4147 | 6286 | 5774 | 3988 |  |
| Age, years | 48.31 (0.33) | 48.16 (0.35) | 46.62 (0.34) | 43.89 (0.44) | <0.001 |
| BMI, kg/m^2^ | 31.06 (0.17) | 30.93 (0.13) | 29.05 (0.12) | 25.73 (0.12) | <0.001 |
| Waist circumference, cm | 106.05 (0.38) | 104.31 (0.30) | 99.30 (0.29) | 90.13 (0.32) | <0.001 |
| HEI-2015 | 43.11 (0.17) | 46.77 (0.21) | 53.35 (0.24) | 60.84 (0.28) | <0.001 |
| Male, n (%) | 2037 (48.50) | 3190 (49.92) | 2934 (50.31) | 2290 (53.82) | 0.006 |
| Race/ethnicity, n (%) |  |  |  |  | <0.001 |
| Non-Hispanic white | 2032 (71.66) | 2681 (69.35) | 2426 (70.30) | 1759 (73.40) |  |
| Non-Hispanic black | 1059 (12.00) | 1537 (11.54) | 1249 (9.75) | 690 (7.28) |  |
| Mexican American | 477 (6.60) | 953 (8.36) | 881 (8.36) | 424 (5.46) |  |
| Others | 579 (9.74) | 1115 (10.75) | 1218 (11.59) | 1115 (13.86) |  |
| Marital status, n (%) |  |  |  |  | <0.001 |
| Married | 1956 (50.99) | 3431 (59.55) | 3266 (60.70) | 2295 (60.62) |  |
| Single | 1834 (39.61) | 2442 (33.95) | 2167 (33.09) | 1481 (33.80) |  |
| Living with a partner | 357 (9.40) | 413 (6.49) | 341 (6.20) | 212 (5.58) |  |
| Education attainment, n (%) |  |  |  |  | <0.001 |
| Under high school | 1317 (24.43) | 1363 (14.83) | 912 (9.46) | 319 (4.22) |  |
| High school | 1222 (32.42) | 1620 (25.20) | 1211 (20.79) | 534 (11.55) |  |
| Above high school | 1608 (43.15) | 3303 (59.97) | 3651 (69.75) | 3135 (84.23) |  |
| Family PIR, n (%) |  |  |  |  | <0.001 |
| <1.3 | 1943 (34.21) | 1923 (19.56) | 1402 (15.38) | 657 (10.02) |  |
| 1.3-<3.5 | 1503 (38.36) | 2523 (37.34) | 2152 (33.47) | 1239 (26.48) |  |
| ≥3.5 | 701 (27.43) | 1840 (43.11) | 2220 (51.14) | 2092 (63.50) |  |
| Current nonsmoking, n (%) | 1571 (35.66) | 4910 (79.74) | 5255 (91.28) | 3902 (98.41) | <0.001 |
| Low-to-moderate alcohol drinking, n (%) | 1283 (32.94) | 4455 (72.58) | 4962 (85.89) | 3827 (95.99) | <0.001 |
| Adequate physical activity, n (%) | 148 (3.74) | 978 (16.34) | 2604 (49.46) | 3502 (90.15) | <0.001 |
| Healthy diet, n (%) | 197 (4.73) | 1394 (19.52) | 3159 (51.97) | 3328 (84.24) | <0.001 |
| Optimal waist circumference, n (%) | 243 (5.30) | 835 (11.82) | 1342 (21.40) | 2237 (53.87) | <0.001 |
| Hypertension, n (%) | 2130 (46.65) | 2913 (42.00) | 2260 (34.89) | 1068 (23.22) | <0.001 |
| Diabetes, n (%) | 963 (17.77) | 1254 (15.46) | 883 (11.04) | 333 (6.19) | <0.001 |

^a^ Continuous variables were expressed as weighted means and standard errors and categorical variables were expressed as numbers and weighted percentages. The sums of percentages may not reach 100%, owing to the rounding of decimals and missing values.

^b^ Characteristics across healthy lifestyle groups were compared with linear regression for continuous variables and logistic regression for categorical variables.

Abbreviations: BMI, body mass index; HEI, Healthy Eating Index; PIR, poverty-income ratio.

# Table S5. Associations of each healthy lifestyle factor with risk of OAB.

| **Healthy lifestyle factor** | **OR (95% CI)** |
| --- | --- |
| Current nonsmoking | 0.82 (0.70-0.95) |
| Low-to-moderate drinking | 0.81 (0.71-0.92) |
| Adequate physical activity | 0.89 (0.79-1.00) |
| Healthy diet | 0.87 (0.78-0.98) |
| Optimal waist circumference | 0.71 (0.61-0.83) |

Data were presented as odds ratio (95% confidence interval). Covariates included in models were shown in the footnote of Table 2, and five lifestyle factors were mutually adjusted for each other.

Abbreviations: CI, confidence interval; OAB, overactive bladder; OR, odds ratio.

# Table S6. Associations of different lifestyle scores consisting of four lifestyle scores with risk of OAB.

| **Score** | **No. of healthy lifestyle factors** | | |
| --- | --- | --- | --- |
|  | **0-1** | **2** | **3-4** |
| Score consisting of smoking, alcohol consumption, physical activity, and waist circumference | 1.00 (reference) | 0.78 (0.68-0.90) | 0.62 (0.52-0.72) |
| Score consisting of alcohol consumption, physical activity, diet, and waist circumference | 1.00 (reference) | 0.76 (0.67-0.87) | 0.67 (0.58-0.78) |
| Score consisting of smoking, physical activity, diet, and waist circumference | 1.00 (reference) | 0.76 (0.66-0.88) | 0.69 (0.59-0.81) |
| Score consisting of smoking, alcohol consumption, diet, and waist circumference | 1.00 (reference) | 0.72 (0.63-0.83) | 0.62 (0.54-0.70) |
| Score consisting of smoking, alcohol consumption, physical activity, and diet | 1.00 (reference) | 0.73 (0.63-0.85) | 0.62 (0.53-0.72) |

Data were presented as odds ratio (95% confidence interval). Covariates included in models were shown in the footnote of Table 2.

Abbreviations: OAB, overactive bladder.

# Table S7. Association of healthy lifestyle score with risk of OAB after redefining the healthy level of alcohol drinking.

| **Variable** | **No. of healthy lifestyle factors** | | | | **Each additional healthy lifestyle factor** |
| --- | --- | --- | --- | --- | --- |
|  | **0-1** | **2** | **3** | **4-5** |  |
| Case/total (%) | 656/2567 (25.56) | 1489/6529 (22.81) | 1227/6556 (18.72) | 529/4543 (11.64) |  |
| Crude model | 1.00 (reference) | 0.77 (0.66-0.91) | 0.59 (0.49-0.72) | 0.37 (0.31-0.44) | 0.75 (0.71-0.79) |
| Model 1^a^ | 1.00 (reference) | 0.68 (0.58-0.81) | 0.52 (0.42-0.64) | 0.37 (0.31-0.44) | 0.75 (0.71-0.79) |
| Model 2^b^ | 1.00 (reference) | 0.77 (0.65-0.92) | 0.65 (0.53-0.79) | 0.57 (0.47-0.69) | 0.85 (0.81-0.90) |

^a^ Model 1 was adjusted for age (<50, ≥50 years), sex (male, female), and race/ethnicity (non-Hispanic white, others).

^b^ Model 2 was further adjusted for marital status (married, others), family poverty-income ratio (<3.5, ≥3.5), education attainment (above high school, high school and below), hypertension (yes, no), and diabetes (yes, no).

Abbreviations: OAB, overactive bladder.

# Table S8. Association of healthy lifestyle score with risk of OAB after propensity score adjustment.

| **Variable** | **No. of healthy lifestyle factors** | | | | **Each additional healthy lifestyle factor** |
| --- | --- | --- | --- | --- | --- |
|  | **0-1** | **2** | **3** | **4-5** |  |
| Case/total (%) | 1169/4147 (28.19) | 1370/6286 (21.79) | 948/5774 (16.42) | 414/3988 (10.38) |  |
| Crude model | 1.00 (reference) | 0.65 (0.56-0.77) | 0.47 (0.40-0.56) | 0.30 (0.26-0.36) | 0.71 (0.68-0.74) |
| PS-adjusted model ^a^ | 1.00 (reference) | 0.75 (0.64-0.88) | 0.62 (0.52-0.74) | 0.53 (0.45-0.63) | 0.83 (0.79-0.87) |

^a^ PS was defined as the probability of being rheumatoid arthritis given a set of covariates, including age (<50, ≥50 years), sex (male, female), race/ethnicity (non-Hispanic white, others), marital status (married, others), family poverty-income ratio (<3.5, ≥3.5), education attainment (above high school, high school and below), hypertension (yes, no), and diabetes (yes, no).

Abbreviation: OAB, overactive bladder; PS, propensity score.

# Table S9. Association of healthy lifestyle score with risk of OAB after imputing missing covariates with multiple imputation.

| **Variable** | **No. of healthy lifestyle factors** | | | | **Each additional healthy lifestyle factor** |
| --- | --- | --- | --- | --- | --- |
|  | **0-1** | **2** | **3** | **4-5** |  |
| Case/total (%) | 1271/4491 (28.3) | 1524/6888 (22.13) | 1048/6366 (16.46) | 454/4290 (10.58) |  |
| Crude model | 1.00 (reference) | 0.66 (0.56-0.77) | 0.47 (0.40-0.55) | 0.31 (0.26-0.36) | 0.71 (0.68-0.74) |
| Model 1^a^ | 1.00 (reference) | 0.64 (0.55-0.76) | 0.47 (0.40-0.56) | 0.35 (0.30-0.41) | 0.73 (0.70-0.77) |
| Model 2^b^ | 1.00 (reference) | 0.74 (0.63-0.87) | 0.62 (0.52-0.73) | 0.55 (0.46-0.65) | 0.83 (0.79-0.88) |

^a^ Model 1 was adjusted for age (<50, ≥50 years), sex (male, female), and race/ethnicity (non-Hispanic white, others).

^b^ Model 2 was further adjusted for marital status (married, others), family poverty-income ratio (<3.5, ≥3.5), education attainment (above high school, high school and below), hypertension (yes, no), and diabetes (yes, no).

Abbreviations: OAB, overactive bladder.

# Table S10. E-values and lower limit of 95% CIs for the association of healthy lifestyle score with risk of OAB.

| **Variable** | **OR (95% CI)** | ***E*-value** |
| --- | --- | --- |
| 0-1 healthy lifestyle factors | 1.00 (ref.) | – |
| 2 healthy lifestyle factors | 0.74 (0.62-0.87) | 2.04 |
| 3 healthy lifestyle factors | 0.62 (0.52-0.73) | 2.61 |
| 4-5 healthy lifestyle factors | 0.54 (0.45-0.65) | 3.11 |
| Each additional healthy lifestyle factor | 0.83 (0.79-0.88) | 1.70 |

Abbreviations: CI, confidence interval; OAB, overactive bladder; OR, odds ratio.

# Table S11. The coefficients of each healthy lifestyle factor in the logistic regression model.

| **Healthy lifestyle factor in the model** | **β coefficient** | **Weighted β coefficient** |
| --- | --- | --- |
| Tobacco smoking (healthy vs unhealthy) | -0.20 | 0.20 |
| Alcohol drinking (healthy vs unhealthy) | -0.21 | 0.21 |
| Physical activity (healthy vs unhealthy) | -0.12 | 0.12 |
| Diet (healthy vs unhealthy) | -0.14 | 0.14 |
| Waist circumference (healthy vs unhealthy) | -0.34 | 0.34 |
| Total | -1.01 | 1.00 |

# Table S12. Association of weighted healthy lifestyle score with risk of OAB.

| **Variable** | **Quartiles of weighted healthy lifestyle score** | | | | **Each additional score** |
| --- | --- | --- | --- | --- | --- |
|  | **Quartile 1 (lowest)** | **Quartile 2** | **Quartile 3** | **Quartile 4 (highest)** |  |
| Case/total (%) | 1606/5751 (27.93) | 917/4439 (20.66) | 1090/6587 (16.55) | 288/3418 (8.43) |  |
| Crude model | 1.00 (reference) | 0.67 (0.57-0.78) | 0.51 (0.44-0.58) | 0.23 (0.19-0.28) | 0.68 (0.65-0.71) |
| Model 1^a^ | 1.00 (reference) | 0.67 (0.58-0.79) | 0.52 (0.45-0.59) | 0.33 (0.28-0.40) | 0.71 (0.68-0.75) |
| Model 2^b^ | 1.00 (reference) | 0.79 (0.67-0.92) | 0.67 (0.59-0.77) | 0.50 (0.41-0.60) | 0.82 (0.77-0.86) |

^a^ Model 1 was adjusted for age (<50, ≥50 years), sex (male, female), and race/ethnicity (non-Hispanic white, others).

^b^ Model 2 was further adjusted for marital status (married, others), family poverty-income ratio (<3.5, ≥3.5), education attainment (above high school, high school and below), hypertension (yes, no), and diabetes (yes, no).

Abbreviations: OAB, overactive bladder.
